# Cumplimiento de las especificaciones en un programa de garantía externa de la calidad. ¿Han tenido impacto los nuevos estimados de variación biológica de la European Federation of Laboratory Medicine (EFLM) en la calidad de los resultados del laboratorio?

**DOI:** 10.1515/almed-2023-0057

**Published:** 2023-10-23

**Authors:** Carmen Ricós, Carmen Perich, Sandra Bullich, Montserrat Ventura, Berta Piqueras, Mariona Panadés, Pilar Fernández-Calle

**Affiliations:** Comité de Programas Externos de la Calidad, Sociedad Española de Medicina de Laboratorio (SEQC^ML^), Barcelona, España; Departamento de Medicina del Laboratorio, Hospital Universitario La Paz, Madrid, España

**Keywords:** garantía externa de la calidad, especificación de la prestación analítica, variación biológica

## Abstract

**Objetivos:**

Los resultados de los programas de garantía externa de la calidad se evalúan frente a especificaciones generalmente basadas en los datos de variación biológica (VB). En este trabajo se pretende comprobar, por un lado, si el cumplimiento de especificaciones varía con la aplicación de nuevos valores de VB y, por otro lado, señalar qué patologías estarían comprometidas debido a una prestación analítica poco satisfactoria de sus mensurandos clave.

**Métodos:**

El material utilizado son los resultados de los programas externos de la SEQC^ML^ desde 2015 hasta 2022. El método es estimar la desviación del resultado individual respecto al valor diana considerado y calcular el porcentaje de resultados que cumplen la especificación pre-establecida.

**Resultados:**

En 97 de los 133 mensurandos el cumplimiento se mantiene entre el 80 y el 100 % de los resultados obtenidos en los dos períodos estudiados. En 23 mensurandos el grado de cumplimiento oscila entre el 51 y el 79 % en los dos periodos. En ALT, AST y sodio el grado de cumplimiento es igual o menor al 50 % de los resultados en el primer período, quedando en este grupo únicamente el sodio en el segundo período.

**Conclusiones:**

Para la mayoría de los mensurandos estudiados el cumplimiento se mantiene independiente de la especificación empleada (SEQC^ML^ o EFLM). Los resultados de ion sodio están por debajo del valor diana, por lo que podrían darse casos de diagnóstico falso de hiponatremia. Los métodos de picrato alcalino no compensado sobreestiman la creatinina, pudiendo ocasionar falsa información de insuficiencia renal.

## Introducción

El control externo de la calidad analítica es una actividad de la medicina del laboratorio, organizada por una entidad externa al laboratorio, que consiste en la distribución de muestras control de forma programada y la evaluación de los resultados obtenidos, según describe la norma ISO/IEC Guide 43 [1]. Tiene un papel de seguimiento de los métodos analíticos reconocido por ISO/IEC Guide 43 [1] y la reglamentación europea actualmente en vigor IVD 2017/746 [2], pone énfasis en la evaluación de la capacidad diagnóstica de las magnitudes del laboratorio [3, 4] y constituye un requisito de la norma ISO 15189 para la acreditación de laboratorios clínicos [5].

La SEQC^ML^ ofrece 17 programas de Garantía Externa de la Calidad (EQA) en ciclos anuales con periodicidad mensual o trimestral, desde el año 1980 [6] y de forma ininterrumpida hasta la actualidad. En el último periodo evaluado (2022) participan 919 laboratorios. Los resultados de los participantes se evalúan frente a una especificación derivada de la variación biológica (VB), siempre que ésta exista.

Las especificaciones derivadas de la variación biológica se habían descrito en 1999 y variaron en 2019, con la publicación de la base de datos de EFLM.

Los objetivos de este trabajo son:

– Comprobar si el cumplimiento de las especificaciones de la calidad derivadas de la VB varía al aplicar los nuevos estimados de la European Federation of Clinical Chemistry and Laboratory Medicine (EFLM), derivados de aplicar una herramienta de lectura crítica y un meta-análisis, sobre la base de los resultados obtenidos en los EQA de la Sociedad Española de Medicina de Laboratorio (SEQC^ML^) durante ocho años.
– Señalar los mensurandos que no cumplen especificaciones, en cuyas prestaciones los laboratorios clínicos y la industria *in vitro* deberían mejorar para evitar errores diagnósticos o de seguimiento y qué patologías estarían más comprometidas debido a una prestación analítica poco satisfactoria.

## Materiales y métodos

El material utilizado es el conjunto de datos de EQA y se centra en los 9 programas de la fase analítica para pruebas que se miden de forma cuantitativa.

Los resultados se obtienen del programa informático de gestión de EQA desarrollado por la SEQC^ML^. El método para tratar estos datos consiste en:

(1) Extraer los resultados individuales de cada laboratorio, de forma anónima, para cada mes (ronda) y cada programa.
(2) Agrupar los resultados: siempre que haya más de 10 resultados aceptados se forma el grupo par (mismo método/instrumento); si no el grupo de métodos (mismo método con distintos instrumentos) y si tampoco se llega a 10 resultados, el grupo lo forman todos los laboratorios.
(3) Detectar y eliminar valores fuera del intervalo delimitado por la media ±3 desviaciones estándar, de forma iterativa, hasta que no quede ningún valor fuera de este intervalo [7].
(4) Estimar la desviación del resultado individual respecto al valor diana considerado en cada programa, expresado en porcentaje de desviación.
(5) Calcular el porcentaje de resultados (percentil) que cumplen la especificación para el error total definida en cada ronda, ciclo y programa.

Para este estudio se consideran 2 periodos. En el primero (2015–2018) el grado de cumplimiento se calculó frente a las especificaciones basadas en la VB elaboradas por la SEQC^ML^ [8] y en el segundo (2019–2022) se hace frente a las de la EFLM [9]. Se considera cumplir la especificación cuando el error total obtenido es igual o inferior al valor de la especificación. Para cuantificar el grado de cumplimiento se toma el valor del percentil más alto que cumple la especificación.

En los programas suero conmutable-bioquímica y suero conmutable-riesgo cardiovascular el valor diana es el valor asignado por un método de referencia certificado. En los restantes programas, el valor diana es la media de los laboratorios del grupo par (método/instrumento) o del método o del global.

Se usan las especificaciones de prestación analítica derivadas de la VB porque permiten controlar el ruido analítico, con lo que la variación encontrada en resultados seriados de un individuo podría ser explicada por la suma de fluctuación fisiológica y la variabilidad analítica [10, 11].

## Resultados

Los programas con los mensurandos incluidos, los criterios de evaluación y las especificaciones utilizadas se muestran en las (Tablas 1–3). Estas Tablas se organizan en función de la categoría de la especificación de VB empleada: óptima, deseable o mínima [12].

El número total de mensurandos de los programas estudiados es de 184. De ellos, 133 se evalúan con respecto a los datos derivados de la VB (Tablas 1–3). Los 51 restantes se evalúan con respecto al percentil 90 (resultados obtenidos en el Programa de la SEQC^ML^ del año anterior) y no forman parte del presente trabajo.

**Tabla 1:** Mesurandos con especificaciones derivadas de la VB óptima.

Mesurando	Programa	Especificación, %	
		SEQC	EFLM
Alfa-amilasa	SUE	7,3	6,6
Alfa-amilasa	SCR	7,3	6,6
Alanina-aminotransferasa	SBQ	13,3	8,0
Alanina-aminotransferasa	SCR	13,3	8,0
Aldosterona	HOR	23,3	21,4
Alfa-fetoproteina	HOR	10,9	17,4
Alfa-fetoproteina	TUM	10,9	17,4
Antígeno carcinoembrionario	HOR	12,4	13,5
Antígeno carcinoembrionario	TUM	12,4	13,5
Antígeno prostático específico total	TUM	ND	8,1
Aspartato-aminotransferasa	SBQ	8,3	6,8
Aspartato-aminotransferasa	SCR	8,3	6,8
Antígeno CA 125	TUM	17,3	8,1
Antígeno CA 15-3	TUM	10,4	10,4
Antígeno CA 19-9	TUM	23,0	18,9
Antígeno CYFRA 21-1	TUM	13,9	13,9
Bilirrubina directa	SBQ	22,3	22,3
Bilirrubina total	SBQ	13,5	12,4
Bilirrubina total	SCR	13,5	12,4
Colesterol	SBQ	4,5	4,3
Colesterol	RCV	NE	4,3
Cortisol	HOR	11,4	16,3
Creatina quinasa	SUE	15,2	11,3
Creatina quinasa	SCR	15,2	11,3
Estradiol	HOR	13,4	8,7
Factor reumatoide	PRO	13,5	6,7
Folato	TUM	19,5	19,5
Folitropina	HOR	10,3	10,6
Fosfato no esterificado	SUE	11,0	5,1
Fracción alfa1-globulina, %	PRO	7,9	ND
Fracción gamma-globulina, %	PRO	8,4	ND
Gamma-glutamiltransferasa	SUE	11,1	9,4
Gamma -glutamiltransferasa	SCR	11,1	9,4
Haptoglobina	PRO	13,6	8,5
Hierro (II+III)	SUE	15,3	15,3
Inmunoglobulina M	PRO	8,4	8,5
Insulina	HOR	ND	15,7
Lactato	POCT	15,2	NE
Lutropina	HOR	14,0	14,2
Prolactina	HOR	14,7	11,9
Proteína C reactiva	PRO	28,3	25,4
Proteína C reactiva ultrasensible	RCV	NE	36,5
Tiroglobulina	TUM	11,0	14,9
Tirotropina	HOR	11,9	13,9
Triglicérido	SUE	13,0	13,7
Triglicérido	RCV	NE	13,7
Troponina T (cuantitativo)	CAR	24,5	24,5
Urato	SUE	6,0	6,0
Urato	SCR	6,0	6,0
Urea	SUE	7,8	8,9

NE, no incluido en programa externo hasta 2021; ND, sin valores disponibles en la base de datos; CAR, marcadores cardíacos; GAS, gases en sangre; HOR, hormonas; PRO, proteínas; SUE, bioquímica básica en suero no conmutable; SCR, suero conmutable con valores de referencia-bioquímica; RCV, suero conmutable con valores de referencia-riesgo cardíaco; TUM, marcadores tumorales.

**Tabla 2:** Mesurandos con especificaciones derivadas de la VB deseable.

Mesurando	Programa	Especificación, %	
		SEQC	EFLM
17-Alfa-OH progesterona	HOR	39,7	35,3
Alfa1-glicoproteína ácida	PRO	16,2	12,4
Androstendiona	HOR	23,5	23,5
Antígeno prostático específico libre	TUM	ND	17,5
Apolipoproteína B	PRO	11,5	11,5
Apolipoproteína B	RCV	NE	11,5
Colesterol de HDL	SBQ	11,3	11,1
Colesterol de HDL	RCV	NE	11,5
Complemento C3	PRO	8,4	7,8
Complemento C4	PRO	16,0	12,1
Creatinina	SBQ	8,9	7,5
Creatinina	SCR	8,9	7,5
Creatina quinasa isoenzima, masa	CAR	16,5	ND
Enolasa específica neuronal	TUM	ND	14,0
Fracción alfa2-globulina, %	PRO	12,6	ND
Fracción beta-globulina, %	PRO	11,7	ND
Ferritina	HOR	16,9	16,9
Ferritina	TUM	16,9	16,9
Fosfatasa alcalina	SUE	12,0	10,6
Fosfatasa alcalina	SCR	12,0	10,6
Globulina fijadora hormonas sexuales	HOR	20,4	17,2
Glucosa	POCT	7,0	6,5
Glucosa	SUE	7,0	6,5
Glucosa	SCR	7,0	6,5
Hemoglobina A_1C_	HbA_1C_	3,0	3,1
Inmunoglobulina A	PRO	13,5	9,8
Inmunoglobulina G	PRO	8,0	7,3
Lactato deshidrogenasa	SBQ	11,3	7,7
Lactato deshidrogenasa	SCR	11,3	7,7
Magnesio	SUE	4,8	4,8
Magnesio	SCR	4,8	4,8
Mioglobina	CAR	26,9	26,9
NT-proBNP	CAR	13,0	13,0
Parathormona	HOR	30,2	20,0
Parathormona	TUM	30,2	20,0
pCO2	POCT	5,7	5,7
Péptido C	HOR	20,8	20,8
Potasio	POCT	5,6	4,8
Potasio	SUE	5,6	4,8
Potasio	SCR	5,6	4,8
Proteína	SCR	3,6	3,5
Proteína	SUE	3,6	3,5
Proteína	PRO	3,6	3,5
Testosterona	HOR	21,0	17,0
Tiroxina libre	HOR	8,7	9,6
Transferrina	PRO	3,8	6,8
Transtiretina (Prealbúmina)	PRO	14,5	14,5
Triiodotironina (T3 libre)	HOR	11,3	9,3
Troponina I	CAR	27,9	27,9

ND, sin datos; NE, no incluido en el programa externo hasta 2022.

**Tabla 3:** Mesurandos con especificaciones derivadas de la VB mínima.

Mesurando	Programa	Especificación, %	
		SEQC	EFLM
25-OH-vitamina D	HOR	NE	30,8
Albúmina	PRO	6,1	5,4
Albúmina	SUE	6,1	5,4
Alfa1-antitripsina	PRO	13,8	9,3
Apolipoproteína A1	PRO	9,1	11,2
Apolipoproteína A1	RCV	NE	11,3 revisar
Beta2-microglobulina	PRO	13,5	9,7
Cadenas ligeras libres Kappa	PRO	NE	12,0
Cadenas ligeras libres Lambda	PRO	NE	12,7
Cadenas ligeras totales Kappa	PRO	12,0	NE
Cadenas ligeras totales Lambda	PRO	NE	12,9
Calcio iónico	POCT	3,1	ND
Calcio total	SUE	3,8	3,8
Calcio total	SCR	3,8	3,8
Ceruloplasmina	PRO	11,9	12,2
Cloruro	POCT	2,2	2,0
Cloruro	SUE	2,2	2,0
Cloruro	SCR	2,2	2,0
Colesterol de LDL (directo)	SUE	17,8	20,5
Colesterol de LDL (directo)	RCV	ND	205
Homocisteína	CAR	23,2	23,2
Lipasa	SUE	27,9^a^	21,3
Osmolalidad	SUE	2,3	2,3
Pseudo-colinesterasa	PRO	ND	14,7
S100	TUM	ND	25,5
Sodio	POCT	1,1	1,0
Sodio	SUE	1,1	1,0
Sodio	SCR	1,1	1,0
Sulfato de dehidroepiandrosterona	HOR	19,6	15,6
Tiroxina total	HOR	10,4	13,0
Triiodotironina total	HOR	9,2^a^	17,4

^a^Evaluado respecto a VB deseable hasta 2018. NE, no incluido en programa externo hasta 2021. ND, sin valores disponibles en la base de datos.

Del grupo de mensurados evaluados frente a la especificación derivada de VB, un 41 % lo hace respecto a la especificación óptima, un 36 % con respecto a la deseable, y un 23 % respecto a la mínima. El porcentaje de mensurandos evaluados frente a la categoría óptima aumenta ligeramente en el segundo periodo, a expensas de las categorías deseable o mínima.

### Cumplimiento de las especificaciones derivadas de la VB

El grado de cumplimiento se cuantifica mediante el percentil de resultados con desviación (respecto al valor diana empleado) igual o inferior a la especificación establecida.

La fuente de las especificaciones aplicadas en nuestros programas fueron las descritas por la SEQC^ML^ [8] en el período 2015–2018 y las de la base de datos EFLM [9] en 2019–2022.

Independientemente de la fuente, en la mayoría de los mensurandos se ha mantenido la misma categoría de especificación (mínima, deseable u óptima) en los dos períodos, con algunas modificaciones:

– En 27 mensurandos se ha pasado de deseables a óptimas, porque los laboratorios han mejorado su rendimiento analítico.

En 10 mensurandos se ha cambiado a especificaciones más laxas (de deseables a mínimas) debido al valor más exigente en la base de datos de la EFLM (triiodotironina total, parathormona, alfa1-antitripsina, complemento C4, apolipoproteína A, lactato deshidrogenasa, lipasa, homocisteina); o bien se ha pasado de la categoría óptima a la deseable porque los laboratorios no conseguían alcanzar la primera en mensurandos con datos sólo contenidos en la fuente SEQC^ML^ (CK-MB y péptido C).

El grado de cumplimiento global, teniendo en cuenta todos los mensurandos evaluados, es prácticamente igual en los dos períodos estudiados (83 % y 84 %). Las Figuras Suplementarias 1–7 detallan estos datos por mensurando para cada programa.

Al observar la evolución del grado de cumplimiento, el total de los 133 mensurandos estudiados se divide en tres grupos:

- En 95 de los 133 mensurandos el grado de cumplimiento se mantiene entre el 80 % y el 100 % de los resultados obtenidos, en los dos períodos estudiados. Es un buen cumplimiento.
- En 23 mensurandos el grado de cumplimiento oscila entre el 51 % y el 79 % en los dos periodos y para 12 no se dispone de datos en el primer período. Es un cumplimiento intermedio.
- En el caso de alanina-aminotransferasa (ALT), Aspartato-aminotransferasa (AST) (programa SCR) y sodio (programas SCR y SUE) el grado de cumplimiento es igual o menor al 50 % de los resultados en el primer período, quedando en este grupo únicamente el sodio en el segundo período. Es un cumplimiento insuficiente.

**Figura 1:**
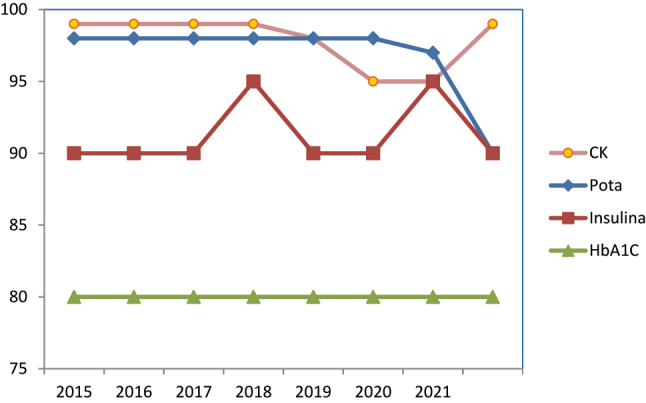
Evolución del cumplimiento de especificaciones. Ejemplos de mesurandos con buena prestación. CK, creatina cinasa; pota, potasio.

Ejemplos de los mensurandos con buena prestación son: potasio en el programa de suero, siempre por encima del 95 % de resultados que cumplen especificaciones; creatina quinasa (CK) e insulina cumplen entre el 80 % y 90 % todos los años, así como HbA_1c_ que mantiene el cumplimiento en el 80 % de los datos aportados a los programas (Figura 1).

**Figura 2:**
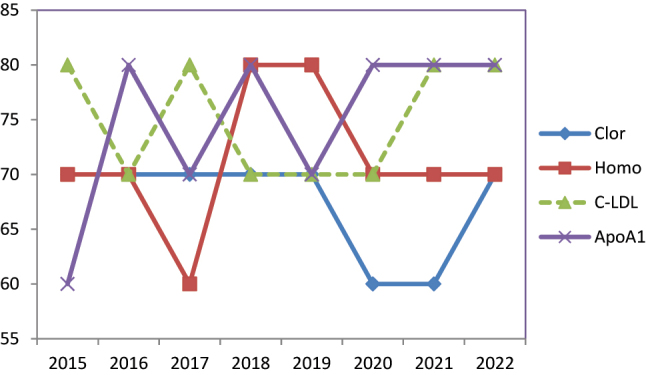
Evolución del cumplimiento de especificaciones. Ejemplos de mesurandos con prestación intermedia. Clor, cloruro, homo, homocisteína; C-LDL, colesterol de LDL; ApoA1, apolipoproteina A1.

Ejemplos de mensurandos con prestación intermedia se muestran en la Figura 2.

**Figura 3:**
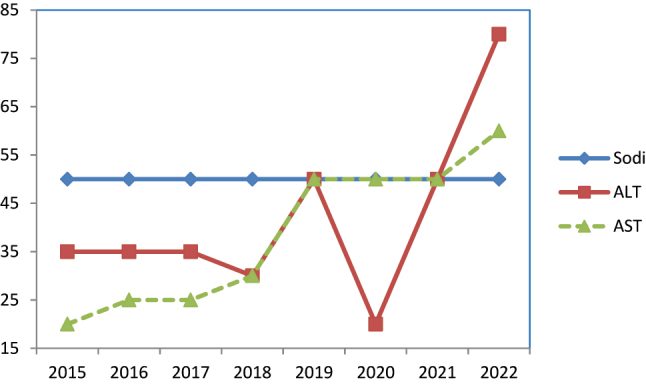
Evolución del cumplimiento de especificaciones. Mesurandos con prestación insatisfactoria. Sodi, sodio. ALT, alanina-aminotransferasa; AST, aspartato-aminotransferasa.

En el grupo de prestación insuficiente se encuentran: sodio (programa de suero); ALT y AST (programa de SCR) (Figura 3).

## Discusión

Los EQA que tienen el objetivo de fomentar la mejora de las prestaciones del laboratorio (como el de la SEQC^ML^) deben usar especificaciones de la calidad exigentes, para incentivar el uso de protocolos de control interno que permitan detectar y prevenir errores que incidan en la información sobre el estado de salud del paciente. Por el contrario, los EQA obligatorios, que tienen como objetivo evitar prestaciones inaceptables, usan límites de aceptabilidad más amplios para que sólo queden fuera un número reducido de “malos” laboratorios, por ejemplo, entre 1 % y 2 % en el caso de CLIA [13].

Actualmente, muchos EQA de distintos países usan especificaciones derivadas de la VB para evaluar a los laboratorios, pasando a ser del 19 % en 1996 al 42 % en 2017 [6], [7], [8], [9], [10], [11], [12], [13], [14]. Incluso un programa de Prueba de Aptitud (*proficiency testing*), como es el de EE.UU. (CLIA), considera ya los datos derivados de la VB como base para establecer sus especificaciones del año 2019 [15, 16].

En este estudio se ha evaluado el rendimiento analítico de los laboratorios participantes en los EQA de la SEQC^ML^ durante el período 2015–2022. Este período abarca las dos fuentes de datos de VB: los cuatro primeros años se evaluaron con respecto a la última actualización de la base de datos de la SEQC^ML^ [8] y los cuatro últimos años con respecto a los estimados revisados y disponibles en la base de datos de la EFLM [9].

Las especificaciones de la EFLM son, en general, un poco más estrictas que las de la SEQC^ML^, habiendo recibido algunas críticas por incitar a los laboratorios a rebajar su nivel de prestación analítica al decidir usar la categoría mínima [17]. Sin embargo, los resultados de este trabajo no reflejan esta circunstancia ni corroboran esta opinión, sino que, en el presente estudio, el grado de cumplimiento se mantiene constante, independientemente de la exigencia de las especificaciones empleadas para evaluar la prestación, para la mayoría de los mensurandos estudiados. De hecho, incluso habiendo cambiado la categoría de la especificación (de mínima a deseable y de ésta a óptima en algunos casos), el nivel de cumplimiento global se mantiene igual.

### Grado de cumplimiento de especificaciones y su evolución

En este trabajo se considera que, si el 80 % o más de los resultados alcanzan las especificaciones, el cumplimiento es bueno. Si lo hacen entre el 50 % y 79 % de los resultados se considera cumplimiento intermedio. Si menos del 50 % de los resultados alcanzan las especificaciones, se considera cumplimiento insuficiente. Este criterio es semejante al utilizado por Panteghini y cols [18] cuando definen especificaciones sobre la base del estado del arte y a los autores les parece adecuado compartir estos criterios de estratificación, aunque estén aplicados a datos distintos.

Algunos mensurandos mejoran su prestación con el paso del tiempo. Probablemente el promover especificaciones derivadas de la VB en los EQA favoreció esta mejora [19], de manera que, como se ha dicho en el apartado de resultados, el porcentaje de cumplimiento global es del 84 %. Así se puede considerar que, en general, las determinaciones cuantitativas de los laboratorios participantes en los EQA de la SEQC^ML^ alcanzan un nivel de calidad excelente.

La SEQC^ML^ ofrece dos programas de nivel 1 (máximo) con análisis replicados de controles conmutables y valores asignados por métodos de referencia certificados [20, 21]. Se trata de los programas suero conmutable con valores de referencia (SCR) y riesgo cardiovascular (RCV), donde el atributo que se evalúa es la estandarización entre prestaciones.

El grado de cumplimiento de especificaciones de los mensurandos incluidos en el programa de SCR es aceptable (77 %), aunque algo inferior al obtenido en el programa de bioquímica básica en suero no conmutable (SUE) (85 %) para los mensurandos comunes. El motivo es que las prestaciones analíticas entre grupos homogéneos están bien armonizadas (programa en suero no conmutable), pero algunos grupos tienen dificultades para alcanzar el valor de referencia certificado (programa de SCR). Los ejemplos más relevantes son:

– AST y ALT, debido al todavía importante número de laboratorios que no incluyen piridoxal-fosfato en el reactivo, obteniendo resultados muy inferiores al valor de referencia [14, 22].
– Calcio, con resultados inaceptablemente inferiores al valor de referencia desde 2015 a 2017 y con resultados satisfactorios desde 2018 a 2021. El motivo es la menor utilización de un calibrador trazable a un material de referencia acuoso (matriz distinta al suero humano), como ya había sido recomendado por Panteghini y Ambruster [23, 24].
– Proteína, para la cual el grado de cumplimiento de especificaciones ha disminuido con el tiempo (del 80–90 % al 65 %). Ello podría ser debido a que un instrumento con muchos participantes (cobas c701 y c702) había obtenido resultados satisfactorios hasta 2019 pero manifestó desviación negativa en 2020 y 2021, sobre todo a concentración superior a 80 g/L, sin que aparentemente hubiera un cambio en el reactivo (Biuret punto final), ni en la trazabilidad analítica del calibrador (NIST-SRM 927). Este hecho pone de manifiesto que es necesario mejorar la colaboración con el fabricante para indagar posibles cambios en la cadena de trazabilidad desde su “masterlot” hasta el calibrador de rutina. Esta misma opinión manifiestan Greg Miller y cols. en un artículo reciente [25]. Este problema no se observa en el programa de SUE porque el valor diana es el promedio de resultados del grupo par y si este grupo par está muy alejado del valor real del mensurando, el rendimiento analítico “real” del laboratorio no es el que reflejan los resultados del EQA.

En el programa de RCV el grado de cumplimiento oscila entre el 84 % y el 95 % de los resultados, en función del mensurando; mostrándose por tanto un buen cumplimiento.

Es importante resaltar el satisfactorio cumplimiento de especificaciones en el programa de SCR porque ocurre en determinaciones sobre material conmutable con el suero humano, que refleja fielmente lo que ocurre al analizar las muestras de pacientes.

A la vista de estos resultados, respecto a los mensurandos con prestaciones analíticas insuficientes para asegurar el uso clínico de estas pruebas, existe una serie de entidades clínicas cuyo diagnóstico o seguimiento podría verse comprometido por el pobre rendimiento analítico de los métodos de laboratorio actualmente disponibles:

– Hiponatremia
– Hipocalcemia
– Evaluación del estado nutricional*-* desnutrición
– Insuficiencia renal

### Hiponatremia

El diagnóstico de hiponatremia se basa en la medida del ion sodio. La especificación deriva de la VB mínima (ET=1,0 %).

En el programa de SCR prácticamente todos los participantes usan el método de potenciometría indirecta. En el primer periodo (2015–2018) cumplían menos del 50 % y a partir de 2019 mejoró el grado de cumplimiento hasta un 60–70 %. Esto puede ser debido a que a lo largo de los años aumenta la utilización del grupo con mejor prestación (cobas c701 y c702). Cabe destacar, sin embargo, que en todos los ciclos los resultados están por debajo del valor de referencia certificado (hasta −2 %), por lo que podrían darse casos de falsos diagnósticos de hiponatremia.

En el programa de SUE, con 890 participantes y con potenciometría indirecta como grupo mayoritario, solo el 50 % de ellos alcanza la especificación en todos los ciclos estudiados.

En el programa de gases en sangre (POCT) (750 laboratorios), donde se usa la potenciometría directa, hay poca dispersión entre grupos (CV inter-laboratorios entre 0,3 y 0,9 %, según el instrumento) en todos los ciclos y por tanto los resultados son similares entre los distintos grupos. Sin embargo, no se pueden comparar con un valor asignado por método de referencia.

Los laboratorios que analicen muestras de pacientes de forma indistinta por sistemas analíticos de potenciometría directa e indirecta, deberían asegurar la intercambiabilidad de los resultados y ser conscientes de la posible tendencia a producir falsa información de hiponatremia.

La industria del diagnóstico *in vitro* debería corregir la posible inexactitud negativa de sus productos, observada en este estudio.

### Hipocalcemia

Los mensurandos de interés son calcio total y calcio ionizado. Para calcio total la especificación deriva de la VB mínima (ET=3,8 %).

En el programa de SCR ya se ha mencionado anteriormente que se obtienen resultados satisfactorios a partir de 2018 hasta 2021. Los métodos más usados son BAPTA calibrador trazable a NIST-SRM 956 (cobas serie c500 y c700) y Arsenazo con la misma trazabilidad (Architect serie c).

Los pocos laboratorios que todavía usan un calibrador trazable a un material de referencia acuoso siguen dando resultados inferiores en un 10 % al valor de referencia certificado. Este grupo podría dar falsos resultados de hipocalcemia, con el consiguiente riesgo de suplementación innecesaria y posible iatrogenia derivada.

Para el calcio ionizado, la especificación deriva de la VB mínima (ET=3,1 %). Este mensurando está incluido en el programa de POCT. El método usado por casi todos los participantes es la potenciometría directa. El valor diana viene dado por el grupo método/instrumento y se mantiene constante la desviación relativa de resultados entre instrumentos. De ello se deduce que mientras un laboratorio no haya cambiado su instrumento analítico, sus resultados son coherentes en el tiempo, aunque en ningún caso se dispone de un valor de referencia certificado de las muestras control distribuidas.

### Evaluación del estado nutricional–desnutrición

El marcador más utilizado para la evaluación preliminar del estado nutricional es la albúmina y la especificación deriva de la VB mínima (ET=5,4 %).

Este mensurando no está incluido en el programa de SCR, por lo que no se dispone de controles con valores asignados por método de referencia.

En el programa de SUE el grado de cumplimiento es constante y alrededor de un 80 %. El motivo de esta armonización es que la mayoría de los laboratorios usan el método verde de bromocresol, con resultados muy similares entre sí. Por el contrario, los usuarios del método púrpura de bromocresol (20 % de laboratorios) obtienen resultados un 60 % inferiores.

En el Programa de Proteínas (PRO) el grado de cumplimiento para albúmina se sitúa en un 77 %. La mayor parte de los laboratorios usan verde de bromocresol y el valor diana viene definido por el mismo instrumento que en el programa de SUE. Sorprendentemente, los pocos usuarios del método púrpura de bromocresol producen resultados algo inferiores al grupo mayoritario (−6 %), pero no tanto como en el programa de SUE. Esta diferencia de comportamiento podría ser debida a la matriz del material control, que en el programa de suero es bovino y en el de proteínas es humano.

Los laboratorios que usan el método púrpura de bromocresol podrían dar falsa información de desnutrición, si no tienen los valores de referencia poblacionales adecuados a su método; o información confusa en un laboratorio clínico en la que se empleen las dos metodologías simultánea o alternativamente (urgencias/rutina).

### Evaluación de la función renal – enfermedad renal crónica

El mensurando clave para el estudio de la función renal es la creatinina sérica, a partir de la cual se estima el filtrado glomerular (FG) a través de diferentes fórmulas, siendo la más recomendada la fórmula CKD-EPI [26]. Las distintas fórmulas existentes, con sus diferentes sensibilidades diagnósticas, contribuyen a aportar mayor variabilidad a la clasificación de los pacientes.

La especificación para la medida de la creatinina sérica deriva de la VB deseable (ET=7,5 %). El grado de cumplimiento está alrededor del 80 % durante los ocho años del estudio. Los métodos enzimáticos (trazables a NIST-SRM 914a y 967a), así como el método de picrato alcalino compensado (trazable a IDMS) producen resultados correctos. Pero los usuarios del método picrato alcalino no compensado producen resultados hasta un 30 % superior a concentraciones entre 50 y 75 μmol/L Éstos, desafortunadamente, aún constituyen aproximadamente la mitad de los participantes en los programas de la SEQC^ML^, conllevando a sobreestimaciones de la cifra de creatinina sérica. Esta sobreestimación se traduce en estimaciones de FG que clasifican a los pacientes en peor estadio de función renal del que le correspondería y que entrañan por tanto un seguimiento, exploraciones complementarias y monitorizaciones innecesarias para el paciente. Estos datos se reproducen tanto en el programa de SCR como en el de SUE por lo que se puede colegir que, lamentablemente, todavía hay demasiados laboratorios en España que usan métodos que sobreestiman la creatinina, a niveles clínicamente relevantes, como para producir falsa información de insuficiencia renal, que conlleva un gasto sanitario innecesario.

## Conclusiones

1. Para la mayoría de los mensurandos estudiados, el grado de cumplimiento de especificaciones se mantiene constante, independientemente de la exigencia de la especificación empleada (SEQC^ML^ o EFLM). Incluso habiendo cambiado la categoría de la especificación (de mínima a deseable y de ésta a óptima en algunos casos), el nivel de cumplimiento se mantiene estable.
2. Algunos mensurandos mejoran su prestación con el paso del tiempo, siendo el grado de cumplimiento global al final del estudio del 84 %. Se puede considerar que, las determinaciones cuantitativas de los laboratorios participantes en los EQA de la SEQC^ML^ muestran buen cumplimiento de especificaciones.
3. Los resultados de ion sodio están por debajo del valor de referencia certificado, por lo que podrían darse casos de diagnóstico falso de hiponatremia. La industria de diagnóstico *in vitro* debería corregir la posible inexactitud negativa de sus productos, observada en este estudio.
4. En la determinación de calcio total los pocos laboratorios que todavía usan un calibrador trazable a un material de referencia acuoso siguen dando resultados inferiores en un 10 % al valor de referencia certificado, pudiendo dar falsos resultados de hipocalcemia.
5. Los métodos de picrato alcalino no compensado sobreestiman la creatinina, pudiendo producir falsa información de insuficiencia renal.

## Supplementary Material

Supplementary Material

## Material Suplementario

Este artículo incluye material suplementario (https://doi.org/10.1515/almed-2023-0057).
