# Supplementary material for: Cumplimiento de las especificaciones en un programa de garantía externa de la calidad. ¿Han tenido impacto los nuevos estimados de variación biológica de la European Federation of Laboratory Medicine (EFLM) en la calidad de los resultados del laboratorio?
Source: Adv Lab Med. 2023 Oct 23;4(4):387–95. [Article in Spanish] doi: 10.1515/almed-2023-0057 (PMC10724878; doi:10.1515/almed-2023-0057)
Supplement: Supplementary file 1 — Supplementary Material [file j_almed-2023-0057_suppl_001.docx]

Figura complementaria 1. Grado de cumplimiento de los mensurados del programa de Marcadores Cardíacos

Figura complementaria 2. Grado de cumplimiento de los mensurados del programa de Gases en sangre-POCT

Figura complementaria 3. Grado de cumplimiento de los mensurados del programa de Hormonas

Figura complementaria 4. Grado de cumplimiento de los mensurados del programa de Proteínas

Figura complementaria 5. Grado de cumplimiento de los mensurados del programa de Marcadores Tumorales

Figura complementaria 6. Grado de cumplimiento de los mensurados del programa de Bioquímica básica en suero no conmutable

Figura complementaria 7. Grado de cumplimiento de los mensurados del programa de Suero conmutable con valores de referencia-bioquímica

**Pie de las figuras complementarias 1-7**

Mensurandos en orden creciente de grado de cumplimiento en el 2º período

**Pie de la figura complementaria 1**

Homo: homocisteina; NT-BNP: péptidos natriuréticos; Tro-I: Troponina I: CKMBm: creatina quinasa isoenzima MB-masa; Miog: mioglobina; Tro-T: Troponina T

**Pie de la figura complementaria 2**

Sodi: sodio; pCO_2_: presión parcial de CO_2_; Ca++: calcio iónico; Clor: cloruro: Gluc:glucosa; Lact: lactato; Pota: potasio

**Pie de la figura complementaria 3**

PTH: parathormona; PepC: péptido C;T3to: triiodotironina total; VitD: 25 OH vitamina D; Andr: androstendiona; T4l: tiroxina libre; T4to: tiroxina total; V-B12: vitamina B12; 17OHP: 17-α-OH progresterona; Est: estradiol; FSH: folitropina;PTH: parathomona; SDHEA: sulfato de dehidroepiandrosterona; FPSA: antígeno prostático específico libre; Aldo: aldosterona; Fola: folato; T3li:triiodotironina libre; Cort: cortisol; Ferr: ferritina; LH: lutropina; Prol: prolactina; CEA: antígeno carcinoembrionario; PSA: antígeno prostático específico; SHBG: globulina fijadora de las hormonas sexuales; AFP: alfa-fetoproteina; Insu: insulina; Test: testosterona; TSH: tirotropina.

**Pie de la figura complementaria 4**

CllK: cadenas ligeras libres kappa; Albu: albúmina; PsColi:pseudocolinestersa; b%: fracción betaglobulina%; ApoA1: apolipoproteina A1; Ceru: ceruloplasmina; Prot: prroteina; a1%: fracción alfaglobulina%; a1-AT: α1-antitripsina; a1-GP: α1-glicoproteina ácida; ApoB: apolipoproteina B; C-C3: complemento C3; CllL: cadenas ligeras libres lambda; Ferr: ferritina; FR: factor reumatoide; a2%: fracción alfa2 globulina %; b2-MG: beta2-microglobulina; C-C4: complemento C4; Hapt: haptoglobina; IgG: inmunoglobulina G; TrFR: transferrina; g%: fracción gammaglobulina%; IgM: inmunoglobulina M; Prealb: prealbúmina; IgA: inmunoglobulina A; PCR: proteína C reactiva.

**Pie de la figura complementaria 5**

S100:Proteinas S100; FPSA: antígeno prostático específico libre; Tirog: tiroglobulina; CA15.3: antígeno CA15.3; TPSA: antígeno prostático específico total; Cyf21.1: antígeno cyfra 21.1; CA125: antígeno CA 125; NSE: enolasa específica neuronal; AFP: alfa-fetoproteina; CA19.9: antígeno CA 19.9+; CEA: antígeno cacinoembrionario.

**Pie de la figura complementaria 6**

Sodi: sodio; Clor; cloruro; Osmo: osmolalidad; C-LDL: colesterol de LDL; Cole: colesterol; Crea: creatinina; Fosf: fosfato no esterificado; Magn: magnesio; Lact: lactato; a-Amilasa: alfa-amilasa; AST: aspartato- aminotransferasa; Lipa: lipasa; Prot: proteína; Liti: litio, LDH: lactato deshidrogenasa; FAL: fosfatasa alcalina; GGT: gamma-glutamil transferasa; Urat: urato; ALT: alanina-aminotransferasa; BilD: bilirrubina directa; Urea: urea; BilT: bilirrubina total; CK: creatina-cinasa; Gluc: glucosa; Hier: hierro (II+III); Trig: triglicérido.

**Pie de la figura complementaria 7**

ALT: alanina-aminotransferasa; AST: aspartato-aminotransferasa; Sodi: sodio; Magn: magnesio; Prot: proteína; Crea: creatinina; a-Amil: alfa-amilasa; Clor: cloruro: Calc; calcio; LDH: lactato-deshidrogenasa; BilT: bilirrubina total; FAL: fosfatasa alcalina; Urat: urato; GGT: gamma-glutamil transferasa; Gluc: glucosa; Pota: potasio; CK: creatina-cinasa.
